# Mine is Earlier than Yours: Causal Beliefs Influence the Perceived Time of Action Effects

**DOI:** 10.3389/fpsyg.2012.00393

**Published:** 2012-10-08

**Authors:** Carola Haering, Andrea Kiesel

**Affiliations:** ^1^Cognitive Psychology Unit, Department of Psychology, Institut fuer Psychologie, Julius-Maximilians-Universität WuerzburgWuerzburg, Germany

**Keywords:** intentional binding, causal belief, causality, temporal order judgments, TOJ, agency

## Abstract

When a key press causes a stimulus, the key press is perceived later and the stimulus earlier than key presses and stimuli presented independently. This bias in time perception has been linked to the intention to produce the effect and thus been called intentional binding (IB). In recent studies it has been shown that the IB effect is stronger when participants believed that they caused the effect stimulus compared to when they believed that another person caused the effect (Desantis et al., 2011). In this experiment we ask whether causal beliefs influence the perceived time of an effect when the putative effect occurs temporally close to another stimulus that is also an effect. In our study two participants performed the same task on connected computers with separate screens. Each trial started synchronously on both computers. When a participant pressed a key, a red and a yellow stimulus appeared as action effects simultaneously or with a slight delay of up to 50 ms. The participants’ task was to judge the temporal order of these two effect stimuli. Participants were either told that one participant caused one of the two stimuli while the other participant seated at the other computer caused the other stimulus, or each participant was told that he/she caused both stimuli. The different causal beliefs changed the perceived time of the effects’ appearance relative to each other. When participants believed they each caused one effect, their “own” effect was perceived earlier than the other participant’s effect. When the participants believed each caused both effects, no difference in the perceived temporal order of the red and yellow effect was found. These results confirm that higher order causal beliefs change the perceived time of an action effect even in a setting in which the occurrence of the putative effect can be directly compared to a reference stimulus.

## Introduction

When an action triggers an effect stimulus, the action and the effect are perceived to be closer to each other in time. For example, when the time of an operant action causing a tone is estimated in relation to a revolving clock hand, the action is perceived later than a non-operant action that does not cause an effect (Haggard et al., 2002a,b; Haggard, 2005). Additionally, tone effects in the operant condition are perceived earlier than tones presented in isolation. Thus, in the operant condition action and effect tone are perceived to be closer in time than actions and tones alone.

This bias in perceived time has been termed intentional binding (IB) because the bias is restricted to conditions in which participants intentionally perform actions. Recent studies demonstrated that key presses and subsequent stimuli are perceived to be closer to each other in time when freely chosen actions produced the stimuli as their effects. However, when the participant’s finger was moved by the key (Wohlschläger et al., 2003a) or the movement of the finger was triggered by a TMS signal (Haggard et al., 2002b) instead of the movement being initialized by the participant him/herself, key presses were perceived earlier and/or tomes were perceived later in these “unintentional” movement conditions, i.e., a reversed pattern of results compared to intentional movement conditions was observed.

Interestingly, IB is not restricted to own actions, but also occurs with observed actions performed by another person (Wohlschläger et al., 2003b). In their intentional observation condition participants judged the time when another person pressed a key. In the unintentional condition participants watched how a key with a rubber hand lying on the key moved downward. The action was perceived to be later in the intentional conditions than in the unintentional rubber hand condition. Thus, the perceived time of the action as a measure of IB is restricted to intentional conditions in which the observer attributes the key presses to an intentional action, even if it is only observed (see also Wohlschläger et al., 2003a). Similarly, the perceived times of actions and effects of a co-actor are closer to each other to a similar degree as those of own actions (Strother et al., 2010).

However, these results are in contrast to a study of Engbert et al. (2007) where no difference in the perceived duration of intervals was found between observed actions of the experimenter and observed key movements with a rubber hand resting on the key. This difference could arise from the different methods used, namely duration estimation and the estimation of the points in time of action and effect. It has been suggested that those methods focus differently on diverging aspects of IB (Humphreys and Buehner, 2009). The estimation of duration relies more on inferential postdictive processes while methods focusing on points in time of action and effect rely on shorter-lived predictive processes. However, this explanation is speculative and has not been directly tested.

To conclude, IB in terms of a shift in the perceived time of action and/or effect occurs for intentional movements, that is for movements that aim at producing a specific effect. IB is not restricted to own intentional actions, but it also occurs for actions of other people that the observer believes to be intentional behavior.

Given that the bias in time perception for actions and contingently following effects depends on own intentional behavior or the belief that a person behaved intentionally, one may assume that IB is stronger for own actions compared to other persons’ actions. For another person’s actions the intention of the actor has to be inferred, while for own actions the intention to act is an inherent predecessor (if not the ultimate cause) of the action. If IB is stronger for own actions compared to another person’s actions, own action effects should be perceived earlier than action effects that are caused or at least *believed* to be caused by someone else.

In line with this reasoning, Desantis et al. (2011) showed that a tone that was caused by a participant was perceived earlier when the participant believed he/she had caused the tone than when the participant believed that the tone had been caused by a key press of another person in the room, a confederate of the experimenters. In their experiment each trial started with the presentation of either the name of the participant or the name of a confederate to inform the participant which one would allegedly be causing a tone effect in this trial. Then, the participant and the confederate pressed a key at approximately the same time. In reality it was always the participant who caused the tone to appear 350, 550, or 750 ms after the key press. After each trial, the participant indicated when he/she had perceived the tone by reporting the position of a revolving clock hand at the moment he/she had perceived the tone. As predicted, participants perceived effects earlier when they believed they had caused the effect compared to the situation when participants believed the confederate had caused the effect, demonstrating that causal belief influenced the perceived time of the effect.

In the current study we aimed at finding further support for the notion that allegedly “own” action effects are perceived earlier than allegedly “another person’s” action effects by using a new design and a psychophysical method to assess time judgments instead of the clock method. In our study two participants performed the experiment simultaneously. Participants were asked to imagine that they were the security officer of a ship and had to save a passenger who fell overboard. The participants’ task was to release either one or two life buoys into the water by pressing a key as quickly as possible. After pressing the key, a red and a yellow life buoy appeared. The temporal order of the two stimuli varied slightly, with a delay of up to 50 ms (varied in 10 ms steps from −50 ms to +50 ms). Half of the participants believed they took part in a shared task and that they caused an “own” single effect, e.g., the red buoy appearing, while the other participant caused the other effect, e.g., the yellow buoy appearing (single effect group). The other half of participants believed as a control group that they always caused one compound effect consisting of the two effect stimuli, i.e., the red and the yellow buoy (compound effect group). That is, in both groups each participant in reality triggered both the red and the yellow life buoy with his/her key press. However, only the compound effect group was veridically instructed that each participant would cause both effects as a compound effect with his/her key press in each trial. The single effect group believed that each participant caused one specific stimulus of the two effects.

To assess the perceived time of action effects, participants performed a temporal order judgment (TOJ) task. That is, they indicated which effect (i.e., the yellow or the red one) they perceived first in each trial. With this design we could directly compare the influence of causal belief on the temporal perception. In the single effect group one effect (e.g., the yellow one) was believed to be the “own” effect while the other effect was believed to be the “other participant’s” effect. So for this group the temporal order of the red and the yellow effect directly represents the order of the “own” and the “other’s” effect. The compound effect group serves as a control group to ensure that not generally the effect of one specific color is preferred regarding temporal order.

Temporal order judgments allow us to estimate IB effects with a psychophysical method. Choosing a psychophysical method also offered the opportunity to analyze not only the perceived time of the effect stimuli relative to each other, but also to compare the temporal resolution of time judgments (Nolden et al., 2012). By using this method we could test not only if the TOJs were biased by the causal belief, but also if participants were less able to distinguish the perceived temporal order of events due to this bias. Regarding the perceived time of effects, we expected that putative “own” effects are perceived earlier than effects that were believed to be caused by another person. When a participant believes he/she caused both effects, the perceived time of those effects should lie in between.

## Materials and Methods

### Participants

Forty-eight students of the University of Wuerzburg (18 male, all right-handed) participated in the experiment due to course requirements. Participants were between 18 and 28 years old (mean 20 years). The data of one additional participant were replaced as he/she did not believe that the experimental computers were actually connected. As the experiment could only be conducted with two participants at a time, the replacing participant took part together with one further participant, whose data were discarded to maintain counter balanced conditions.

### Apparatus and stimuli

The experiment was run on two standard PCs equipped with 17″ CRT screens. The PCs were connected via the computers’ parallel ports to synchronize the beginning of each trial. Stimulus presentation and data collection were accomplished with the software package E-Prime2 (Schneider et al., 2002).

**Figure 1:**
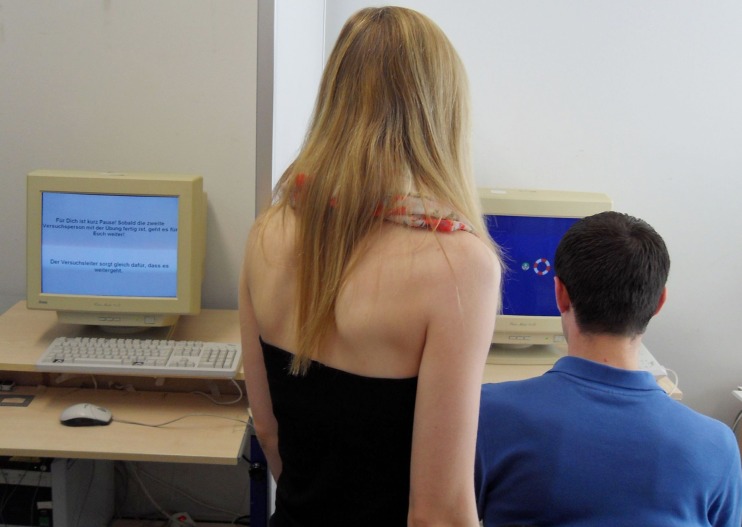
**Experimental layout**. The two computers were connected via parallel port. The situation depicts an instruction block in the single effect group: The right participant performs key presses always triggering his “own” effect, here the red life buoy, while the left participant watches. Earlier the right participant had watched the left participant triggering her “own” effect, the yellow life buoy. After the instruction phase each participant performed the task on his/her computer separated by the divider wall. Throughout experimental trials both participants wore headphones delivering white noise to ensure they did not hear button presses.

The experimental setup is shown in Figure 1. The two screens were placed side by side (distance ca. 1.5 m) with a divider wall in between to ensure that participants only saw their own screen. During the main part of the experiment participants sat in front of their screen. To avoid the participant hearing the other participant pressing the key, both participants wore Vic Firth SIH1 isolation headphones. The experimenter stayed in the room throughout the entire experiment to ensure that participants did not communicate with each other.

Stimuli were presented on an avy blue background. All messages were printed in white. We used a white fixation cross extending 0.7 cm. The imperative stimulus was the head of a person wearing a swim cap (diameter 1.9 cm) that appeared in the middle of the screen, described as a passenger who fell overboard. The targets for the temporal order task were a red and a yellow life buoy (diameter 3.9 cm) appearing 2 cm left or right of the center of the screen.

### Procedure

In each session two participants took part and were either both assigned to the single effect group or both to the compound effect group. Where not stated otherwise, the procedure was the same for both groups. All participants were asked to imagine they were a security officer of a ship who has to save a passenger who repeatedly falls overboard. The participants’ task was to release one (single effect group) or two life buoys (compound effect group) by pressing the left mouse button as fast as possible. After pressing the key, the two life buoys appeared with a slight temporal delay (see trial structure described below).

In the single effect condition, each participant was told that he/she was in control over one of the two life buoys on both computers while the other participant controlled the other life buoy via the connecting cable. One participant was told to control the red life buoy (single red effect condition) and the other participant was told to control the yellow life buoy (single yellow effect condition). In the compound effect group, both participants were told to control both buoys on their own computer. The connection of the computers was explained to ensure that the experiment ran synchronously for both participants in the compound effect group. As each participant caused both effects on his screen, actually in both groups only the starting time of each trial was synchronized.

To improve the credibility of the group-specific instructions regarding who caused which life buoy to occur, participants performed an instruction phase before the main experiment. First, each participant was informed by written instructions that he/she caused either the red buoy, the yellow buoy, or both buoys to appear. Each of the two participants then performed an instruction block while the other participant stood behind and watched (see Figure 1). In the single effect group only the participant’s “own” effect appeared randomly on the left or right side of the “drowning” passenger. In the compound effect group always both buoys appeared with the assignment of color to side of the screen counterbalanced within participants. After the first participant had completed the instruction block, it was his/her turn to watch the second participant accomplishing the instruction block.

**Figure 2:**
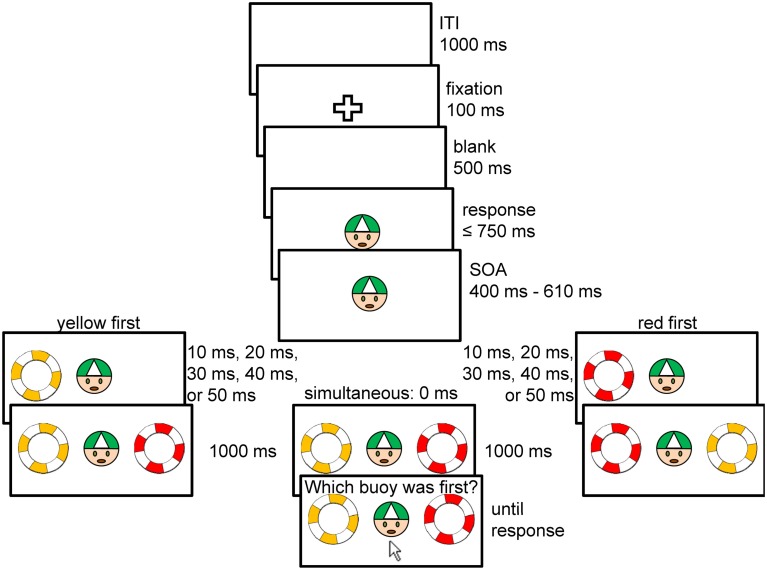
**Schematic trial procedure of the temporal order judgment (TOJ) task**. Effect stimuli (the life buoys) were presented simultaneously or separated by a delay of 10–50 ms. TOJs were given by clicking on the life buoy the participant judged to have appeared earlier. The location of red and yellow stimuli on the screen was counterbalanced within participants. For better readability in the figure the background color is shown in white instead of blue in the experiment and texts are printed in black instead of white.

During the rest of the experiment participants wore isolation headphones and heard constant white noise. The volume of the white noise was adjusted so that participants did not hear the sounds caused by the mouse clicks. Each trial started with the fixation cross presented centrally for 100 ms (for a schematic sketch of experimental trials see Figure 2). After a blank of 500 ms the imperative stimulus, the passenger, followed. The participants’ task was to press the left mouse button as quickly as possible in response to the passenger’s appearance. When the participant pressed the button within the time limit of 750 ms the first of the two effects (life buoys) appeared after a variable interval of between 400 and 610 ms after response onset. The second effect appeared either at the same time (i.e., separated by a delay of 0 ms) or after 10, 20, 30, 40, or 50 ms. We will here after refer to these delays as temporal distance of the yellow effect in relation to the red one, that is, positive delays (10, 20, 30, 40, and 50 ms) indicate that the red effect appeared first, and negative delays (−50, −40, −30, −20, and −10 ms) indicate that the yellow effect appeared first.

After both effects were visible for 1000 ms, participants were asked to indicate which buoy appeared first (the German words “Welcher Rettungsring war zuerst?” appeared above the stimuli). For this TOJ task, the mouse cursor appeared 4.5 cm below the passenger and the participant had to click on the buoy he/she believed appeared first. After clicking on a buoy, all stimuli and the mouse cursor disappeared. The next trial started 1000 ms after both participants had clicked on a buoy.

If a participant did not press the mouse button within 750 ms after the imperative stimulus (the passenger) appeared, the passenger disappeared, and an error message reminded the participant to respond as quickly as possible to save the passenger from drowning (“Bittereagierenach Erscheinen des Passagiersim Wasser so schnellwiemöglich, sonstertrinkter!”). Participants had to acknowledge this message by clicking on a check box labeled “Ok!” to end the trial. This time limit was introduced to avoid very slow responses, because participants could easily realize that very slow responses did not, contrary to the instructions, always cause the “own” effect to appear later than the “other’s” effect.

We included some reminder trials without TOJs in which only one effect occurred to remind participants who controlled which effect. In the single effect groups the “own” effect was accompanied by the message “Diesmal war der andereim Vergleichzulangsam. Du hast den Passagiergerettet!” (German for “This time the other participant was in comparison too slow. You saved the passenger!”). The allegedly “other’s” effect was accompanied by the message “Diesmalwarst Du im Vergleichzulangsam. Der andere hat den Passagiergerettet!” (German for “This time you were slower. The other participant saved the passenger!”). In the respective trials in the compound effect group the message always read “Aufgrundeinertechnischen Fehlfunktionistnure in Rettungsring ins Wassergefallen!” (German for “Due to a technical fault only one life buoy fell into the water”). In both groups the message had to be acknowledged with a click on a check box labeled “Ok!”

The two instruction blocks comprised 20 trials each, resulting in an instruction phase comprising of 20 self-performed and 20 observed instruction trials. After the instruction phase, participants performed 26 practice trials that included all trial types that would be in the main experimental blocks to ensure that participants understood all tasks. Six experimental blocks with 48 trials each followed. In each block, each delay (−50, −40, −30, −20, −10, 0, 10, 20, 30, 40, and 50 ms) was repeated four times. In addition, there were four reminder trials per block in which only one buoy appeared (red or yellow presented at the left or right side). The temporal and spatial order of the effects’ appearance was counterbalanced within participants. Each effect appeared equally as often on the left side as on the right side of the screen.

Before debriefing at the end of the experiments we asked participants separately to describe their task and why the computers’ were connected. All but one (the excluded participant) described the experiment as instructed and did not suspect the instructions to be false.

### Data analysis

In each experimental trial participants indicated whether the yellow or the red effect appeared first. To analyze whether the “own” effect is perceived earlier than the “other’s” effect and to compare TOJs with the compound effect group, we made the arbitrary decision to analyze how often the yellow buoy was perceived earlier than the red buoy^1^, i.e., for each participant and delay we calculated the proportion of “yellow first” responses. Based on this analysis, we expected that participants who believed they caused the yellow effect to perceive the yellow effect earlier than participants who believed they caused the red effect. Furthermore, participants in the compound effect group were expected to perceive the yellow effect later than participants who believed they caused the yellow effect, but earlier than participants who believed they caused the red effect. About 3.9% of all planned TOJ trials were stopped before any effect appeared because the participants did not respond within 750 ms.

We fitted logistic functions to the “yellow first” responses using the psignifit toolbox (Wichmann and Hill, 2001) for MATLAB. From each fitted function we calculated the 50%-value of the function, the Point of Subjective Simultaneity (PSS). This value represents the temporal delay between the yellow and the red effect that results in the participant not being able to discriminate the order of the two stimuli and thus has to guess, resulting in 50% “red first” and 50% “yellow first” responses. When the yellow buoy is perceived earlier than the red buoy, the PSS is larger than zero, indicating that a yellow buoy that occurs x ms after a red buoy is perceived as occurring simultaneously with the red buoy. In contrast, when the red buoy is perceived earlier than the yellow buoy, the PSS is smaller than zero because the yellow buoy that appears × ms before the red buoy is perceived as occurring simultaneously with the red buoy.

We also calculated the difference limen (DL) as the difference between the 75% and the 25% score of the function divided by two. The DL is a measure for the steepness of the function and indicates the temporal resolution of the judgments of each participant. The higher the temporal resolution of judgments, the more consistent a participant is in his/her judgments regarding each delay, resulting in a steeper function and thus a smaller DL.

## Results

We conducted ANOVAs on the PSS and the DLs including the between-subjects factor type of causal belief (single yellow effect, single red effect, compound effect).

**Figure 3:**
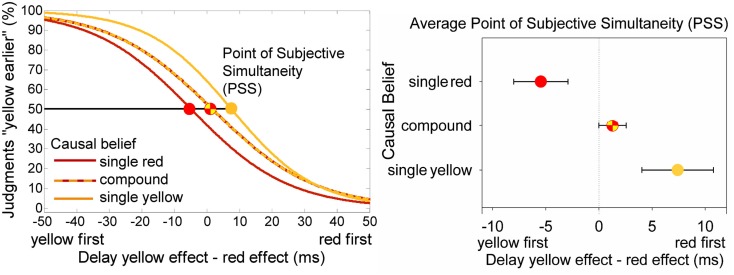
**Results of prototypical participants (left) and group means for the PSS (right)**. On the left fitted functions of three prototypical participants from the single effect yellow and red group (colors according to group name) and the compound effect group (dashed line). The group means of the points of subjective simultaneity are shown in the bar chart on the right. Error bars depict standard errors.

The ANOVA on the PSS revealed differences between causal belief conditions, *F*(2,45) = 6.86, *p* = 0.003, $\eta_{p}^{2}$ = 0.234 (see Figure 3). When participants believed they caused the yellow effect (single yellow effect), the yellow effect would have to appear 7.4 ms after the red effect for them to be perceived simultaneously (i.e., the PSS was 7.4 ms). When participants believed they caused the red effect (single red effect), the red effect would have to appear 5.4 ms after the yellow effect for them to be perceived simultaneously (i.e., the PSS was −5.5 ms). When participants believed they caused both effects (compound effect), the yellow effect would have to appear 1.2 ms after the red effect for them to be perceived simultaneously.

*Post hoc* *t*-tests revealed that each single comparison was significant, i.e., PSS for the single yellow effect group was larger than the PSS in the compound effect group (7.4 vs. 1.2 ms), *t*(34) = −2.06, *p* = 0.047, and it was larger than the PSS in the single red effect group (7.4 vs. −5.5 ms), *t*(22) = −3.04, *p* = 0.006. In the single red effect group the PSS was smaller than in the compound group, *t*(34) = 2.64, *p* = 0.012.

The ANOVA on the DLs revealed no difference between groups, *F*(2,45) = 0.48, *p* = 0.622, $\eta_{p}^{2}$ = 0.021. DLs amounted to 25.9 ms in the single yellow effect group, to 23.8 ms in single red effect group, and to 22.0 ms in the compound effect group.

## Discussion

In this study, we aimed at investigating whether different causal beliefs about who causes one of two effect stimuli influence the perceived temporal order of these effect stimuli. Participants who believed they caused only one of two effect stimuli perceived their “own” effect earlier than the “other’s” effect. This confirms the assumption of stronger IB for allegedly “own” effects than for effects that are believed to be caused by another person. The “own” effect was also perceived earlier than the effect of the respective color in a group of participants who believed they caused both effects as compound effect. Thus, we can rule out that one of the effects was generally perceived earlier than the other due to any stimulus features.

To measure time perception for “own” effects, we applied TOJs. TOJs have recently been shown to be a useful method to measure the IB effect using a psychophysical method (Cravo et al., 2011). Choosing this psychophysical method has at least two advantages. First, in contrast to the clock paradigm, TOJs allow us to directly compare the temporal order of a putative effect and another stimulus within one trial. This let us directly assess the time perception of the “own” and the “other’s” effect instead of deducing temporal order from time estimations in different trials in relation to the revolving clock hand.

Second, TOJs enable us to analyze not only the perceived time (the PSS) of the effect stimuli, but also to compare the DLs of time judgments as a measure of the temporal resolution of time judgments (Nolden et al., 2012). Importantly, DLs did not differ between the single effect group and the compound effect group. Applying this method enabled us to rule out the possibility that the manipulation of the causal belief influenced temporal resolution because, for example, of changing difficulty level of the task. Instead, the belief manipulation added a constant difference to temporal estimations, but left the overall consistency in TOJs unchanged. Thus, TOJs were biased by the causal belief, but this bias did not affect the reliability of the TOJ.

Taken together, our result that “own” effects are perceived earlier strengthens and extends the recent finding of Desantis et al. (2011), who showed that an effect tone is perceived earlier in trials in which the participant believed he/she caused the tone compared to other trials when the participant believed that another person caused the tone. Here the information who would cause the effect tone in the next trial participants could have changed the level of participants’ arousal or motivation in trials in which they knew they would cause a tone with their key press compared to trials in which they knew they would press a key, but hear another person’s effect. In our study, participants compared the perceived time of the “own” and the “other’s” effect relative to each other within each trial. So the participants’ belief that they produced one specific effect remained constant throughout the experiment. This enables us to exclude any possible explanation based on trial-by-trial differences for differing time judgments between “own” and “other’s” effects. Instead causal belief influences the perceived time of action effects on a stimulus-specific level. In addition, assessing the DLs of time judgments enables us to rule out that the temporal resolution differs depending on the instruction to cause one or two effects. To sum up, our study fosters the conclusion that IB is stronger for allegedly “own” action effects than for action effects that are attributed to another person’s action.

The influence of causality and causal beliefs on IB has been discussed from the time the IB effect was first described (see Moore and Obhi, 2012 for a recent review). Eagleman and Holcombe (2002), for example, discussed whether the temporal attraction between action and effect was the counterpart of larger perceived causality between cause and effects the closer the effect appears after the cause (Hume, 1739; Michotte, 1963). Similarly, IB has been discussed as a process that supports the feeling of agency, i.e., the perceived causal control over one’s action effects. Interestingly, agency and IB have been found to be correlated only when both measures are collected within one trial, but not when they are measured in different trials (Ebert and Wegner, 2010; see also Obhi and Hall, 2011). Nevertheless, there is evidence that IB depends on causal beliefs because IB effects occur for action effects, but not for effects caused by observed non-agentic sources (Wohlschläger et al., 2003a,b; Cravo et al., 2009). Furthermore, IB in terms of a later perception of the action is restricted to cases where the causal relation between action and effect is highly reliable in terms where the effect follows the action with high contingency (Moore and Haggard, 2008; Moore et al., 2009).

Recently, the impact of causality on IB has been demonstrated even more convincingly. Dogge et al. (2012) observed IB even in the absence of a voluntary action. In that study, the effect of an involuntary passive key press was perceived shifted toward the key press when participants believed that the passive key press caused the effect. In contrast, when no causal belief instruction was given about a causal relation between key press and effect tone, there was no shift in the perceived time of the effect. The authors assume that the predictive thought of the effect (cf. Wegner and Wheatley, 1999) before the passively induced key press leads to an increased level of perceived control and thus to a shift in the perceived time of the effect when the movement was believed to cause the effect, even in the absence of a voluntary movement. This shift in the perceived time of the effect is smaller after involuntary compared to voluntary key presses, but it shows that even in the absence of an intended movement the causal relation between the movement and the effect is sufficient to induce a certain degree of IB.

Further support of a relation between causal belief and IB is evidenced in a study of Buehner and Humphreys (2009). Their participants heard two tones and were asked synchronize two key presses to the two tones. In a non-causal condition the second tone followed the first after a fixed interval. In a “causal condition” the second tone was caused by the first key press and thus occurred after a fixed interval after the action (the same interval as in the non-causal condition). Actually, participants timed the two key presses in relation to the times of the tones differently in the two conditions, suggesting that they perceived the action and effect to be closer in time in the causal condition as suggested by IB (see Buehner and Humphreys, 2010 for similar results on causal relations between spatial stimuli). However, in this experiment causality was manipulated in that there were physical differences such as different time intervals in the causal and non-causal conditions. In our study there were no physical differences between “own” effects and “other’s” effects across participants confirming that it is actually the causal belief alone that changed the perceived time of action effects.

Interestingly, the conclusion that “own” effects are perceived earlier than others’ effects seems to be contradicted by recent results reported by Obhi and Hall, 2011; for similar results see also Strother et al., 2010). They investigated IB in a social situation, in which two participants performed a task together on one computer with one shared key. In each trial, one participant triggered a tone by pressing the key and the other participant was to respond by pressing the same key as quickly as possible after the key was pressed. Two-hundred milliseconds after the first key press a tone effect occurred. Each participant then judged who they believed had caused the effect (the actor) and the time of the actor’s key press. In this study, the IB effect was not reduced when the participant was the responder and thus judged the time of the actor’s, i.e., another person’s, key press and effect than when the participant was the actor himself/herself. That is, time judgments for the action and the effect were the same, regardless of whether the participant believed that the other participant caused the effect (and thus judged the observed action of the actor) or whether the participant believed himself/herself to be the actor (and thus judged the time of his/her own action).

To resolve this contradiction we suggest that there is a critical difference between the experimental setting of Desantis et al. (2011) and our setting on the one hand, and between the experimental setting of Obhi and Hall (2011) on the other hand. In Obhi and Hall’s study participants were instructed to cooperate on the experimental task. In contrast, in the study of Desantis et al. participants performed the task on their own, and in our study no cooperation was needed because one life buoy would be sufficient to save a swimmer’s life. This fits well with Obhi and Hall’s, 2011, p. 655) suggestion that participants might form “a ‘we’ identity” in the shared task. Even if not directly expressed in the instructions, our task implied a competitive rather than a cooperative situation as only one participant, probably the faster, will complete the task.

The instruction to cooperate on a single task might be the reason that participants showed a similar amount of IB for own and observed actions and effects in Obhi and Hall’s (2011) study. Another line of research, the so-called “social Simon-effect,” demonstrates that in cooperative settings, participants integrate the intention of another person into their own task set (Sebanz et al., 2003; Knoblich and Sebanz, 2006; Dolk et al., 2011; Liepelt et al., 2011). For example, when two participants share a Simon task, that is, one participant responds to green targets by pressing a left key and another participants responds to red targets by pressing a right key, performance is influenced by the location of the target. A participant who responds with the left key responds more slowly when the target stimuli occurs on the right (incompatible) side of the screen than when the target occurs on the left (compatible) side of the screen. In contrast, when one participant performs his/her half of the task alone (which is actually a Go-NoGo task, e.g., respond to green targets, do not respond to red targets), the compatibility effect regarding the location of the target and the response key is heavily reduced. Based on this evidence, we assume that participants adopt the intention of the other participant more strongly when participants cooperate on a task than when they infer from information given on screen that the effect they perceive will be caused by another person performing the same task at the same time (as in the study of Desantis et al., 2011) or when they compete on a task (as in our study). This assumption could explain why on the one hand Obhi and Hall (2011) found in a collaborative situation the size of IB in terms of the perceptual shift of actions and effects toward each other was independent of whether actions and effects are attributed to the own action or the action of an observed participant. On the other hand the assumption would also explain why in non-collaborative situations both Desantis et al. (2011) and we observed stronger IB in terms of an earlier perception of the “own” compared to “another person’s” effect. However, this is a *post hoc* hypothesis and future studies are needed to investigate how cooperation vs. competition changes the perceived time of another person’s action effects.

## Conflict of Interest Statement

The authors declare that the research was conducted in the absence of any commercial or financial relationships that could be construed as a potential conflict of interest.

## Footnotes

^1^As in the compound effect group, because no effect is assigned as “own” effect we cannot analyze according to the “own” and the “other’s” effect. Choosing the “yellow first” responses was arbitrary, but because each response was a discrete decision between “yellow first” and “red first,” the results would have been equal with the opposite sign if we had chosen “red first” responses.
